# Lumbar disc herniation in three patients with cystic fibrosis: a case series

**DOI:** 10.1186/1752-1947-5-440

**Published:** 2011-09-06

**Authors:** Christian Denne, Anna E Vogl-Voswinckel, Harald Wurmser, Marc Steinborn, Manfred Spaeth, Armin Gruebl, Stefan Burdach

**Affiliations:** 1 Department of Pediatrics, Kinderklinik München Schwabing, Klinikum Schwabing StKM GmbH and Klinikum Rechts der Isar (AöR) of the Technical University (TU) München, Munich, Germany; 2 Department of Pediatric Radiology, Institute for Diagnostic Radiology, Klinikum Schwabing StKM GmbH, Munich, Germany; 3 Department of Neurosurgery, Klinikum Schwabing StKM GmbH, Munich, Germany

## Abstract

**Introduction:**

To date, lumbar disc herniation has not been reported in the context of cystic fibrosis even though back pain and musculoskeletal problems are very common in patients with cystic fibrosis.

**Case presentation:**

We report on three patients with cystic fibrosis who experienced lumbar disc herniation in the course of their disease at ages 19 to 21 years (a 22-year-old Caucasian man, a 23-year-old Caucasian man, and a 21-year-old Caucasian woman). Our third patient eventually died because of her deteriorated pulmonary situation, which was influenced by the lumbar disc herniation as it was not possible for her to perform pulmonary drainage techniques properly because of the pain.

**Conclusions:**

Lumbar disc herniation can lead to a vicious cycle for patients with cystic fibrosis as it may promote pulmonary infections. This report highlights the need to investigate patients correctly.

## Introduction

Cystic fibrosis (CF) is a lethal chronic pulmonary disease with recurrent acute infective exacerbations, inevitably leading to progressive bronchiectasis and combined ventilatory disorders [1]. With the improved survival rates in recent decades, CF may also be commonly associated with relevant painful conditions caused by complications of the illness and also by therapy itself, which may cause additional suffering [2,3]. Back pain is a chief complaint in patients with cystic fibrosis [4]. It is often due to vertebral fractures as a consequence of a decreased bone mineral density due to use of steroids [5]. Rheumatoid arthritis, spondyloarthropathies, and posture abnormalities such as kyphosis have also been reported in association with CF [6]. Whereas many papers focus on pain in patients with CF, studies on lumbar disc herniation (LDH) in patients with CF have not been reported to date [7].

## Case presentation

We report on three patients with clinically symptomatic and radiologically proven LDH from our CF clinic. The CF clinic is part of the Department of Pediatrics. The age structure of patients (children, adults) is mixed. In all, 24 of a total of 46 patients from the clinic were older than 18 years according to the Cystic Fibrosis Ambulance System (CFAS) data. The mean age at diagnosis of LDH was 20.5 years. The point prevalence of LDH was 6.5% in our clinic in the year of the case presentation, with respect to all patients. With respect only to adults, the point prevalence was 12.5%. One of these three patients had a neurosurgical decompression operation. Lung function parameters (as shown by forced expiratory volume in one second (FEV1) and forced vital capacity (FVC)) were variable. Family history for LDH was positive in two patients.

### Case 1

**Figure 1:**
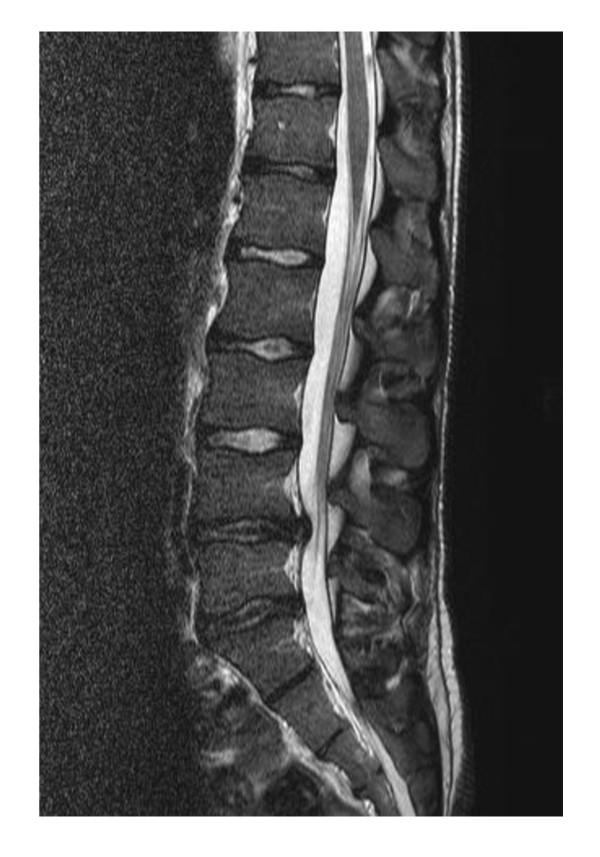
**A sagittal T2-weighted MRI image of the lumbar spine showing a centrally located disk herniation at L4/L5 and to a lesser degree at L5/S1**. Note the decreased signal of the L4/L5 and L5/S1 disks, indicating decreased disk hydration.

**Figure 2:**
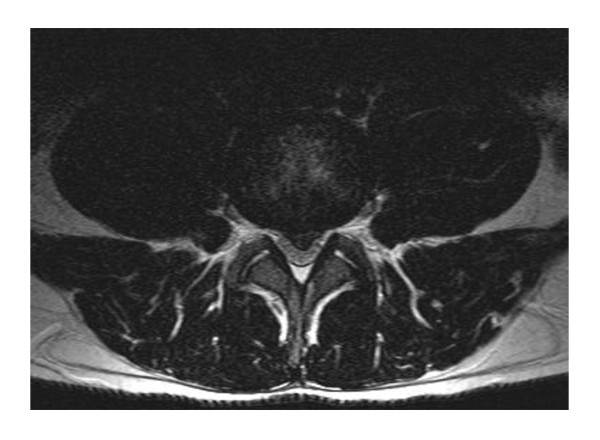
**The corresponding axial T2-weighted MRI image at L4/L5 demonstrates the centrally herniated disk material narrowing the spinal canal**.

Patient 1 was a 22-year-old athletic and muscular Caucasian man (delta F 508 homozygous, *Pseudomonas *positive) with good lung function parameters (Table 1), in whom LDH was diagnosed at an age of 21 years. He had experienced recurrent back pain, predominantly lumbalgia, for about two years before the diagnosis of LDH. It was treated with non-steroidal anti-inflammatory drugs (NSAIDs) by our patient. Two weeks before the LDH diagnosis was made via lumbar nuclear magnetic resonance (NMR) scans, his lumbalgia increased sharply, finally with inguinal pain radiation. Our patient held his excessive physical training responsible for the motion-dependent pain, especially on hip flexion. After a non-operative approach with physiotherapy at first, a decompression operation was then performed because of increasing pain (maximum 10/10 on visual analog scale, opioid dependent) approximately five months after LDH was diagnosed using NMR. For the first two days following the operation, our patient needed nasal oxygen of 1L per minute via prongs and our patient had lumbalgia for about three months after the operation. Intensive physiotherapy was performed post-operatively. No chest infection (pulmonary exacerbation) occurred in the recovery period. His bone density was not examined. Figure 1 and 2 show two representative NMR slides from patient 1 with LDH in lumbar vertebra 4/5 (L4/L5) and L5/sacral vertebra 1 (S1).

**Table 1:** Data from our three patients with cystic fibrosis with lumbar disc herniation (LDH)

**Patient no**.	Age at case presentation	Age at diagnosis of LDH (imaging)	Localization of LDH	Neurosurgical operation	Positive family history of LDH	Use of opioids	Use of NSAIDs	Forced expiratory volume (FEV1)	Vital capacity (VC)
1	22	21	L4/L5 median, L5/S1 median	Yes	Parent	Yes	Yes	3.98L (89.7%/norm)	5.10L (92.6%/norm)

2	23	19	L5/S1 left mediolateral	No	Parent	No	Yes	2.53L (61.1%/norm)	4.48L (88.1%/norm)

3	21	21	L4/L5 left paramedian; L5/S1 left paramedian	No	No	Yes	Yes	0.99L (30.7%/norm)	1.67L (45.6%/norm)

Total number of patients was 46; 24 of these patients were 18 years of age or older. Patient 3 died eight months after diagnosis of LDH.

NSAID = non-steroidal anti-inflammatory drug.

### Case 2

Patient 2 was a 23-year-old Caucasian man (delta F 508 homozygous, *Pseudomonas *positive) who first presented nine years before the date of the documented case to an ambulant orthopedist with acute back pain for three days. A lumbar X-ray was performed which showed hyperlordosis. His bone density was not examined. No special therapy was initiated. About five years before the date of his most recent presentation, our patient had an NMR scan; LDH was suspected as he presented with lumbalgia with left-sided pain radiation. After the diagnosis of a mediolateral LDH at L5/S1 was established, subsequent intermittent episodes of low back pain have been well treated by our patient with muscular training in a fitness center and NSAIDs. No control spinal imaging has been performed so far. His lung function did not change relevantly after the LDH diagnosis. Our patient suffers from allergic asthma (expiratory flow limitation in lung function) comorbidity and recurrent episodes of allergic bronchopulmonary aspergillosis (ABPA), for which he currently needs prednisolone in doses of 5 to 10 mg/day. Bone fractures have not been diagnosed to date.

### Case 3

Patient 3 was a 21-year-old Caucasian woman with an end-stage obstructive and restrictive ventilation disorder (Table 1), who presented to our facility with severe lower back pain, left-sided ischialgia and paresis of the feet and toe extensor muscles as well as paresthesia of the dorsum pedis and toes corresponding to dermatome L5 for one week. A lumbar NMR scan showed a paramedian LDH at L4/5 with compression of nerve root L5 and a paramedian LDH at L5/S1 touching nerve root S1. A decompression operation was not performed despite the neurosurgeons' recommendations as our patient refused because of the fear of prolonged ventilation and respirator dependency after operation in the light of her severely impaired lung function. In spite of intensive physiotherapy, full mobilization took several weeks and inspiration was impaired by the pain, so that it was not possible to perform respiratory physiotherapy to its full extent. The paresis disappeared under physiotherapy but the pain did not fully disappear, needing NSAIDs and opioids. A course of intravenous antibiotic therapy was necessary at one, four and five months later. Her lung function parameters declined further and home oxygen therapy was started three months after LDH diagnosis, with 3 to 6L of O_2 _per minute. From six months after LDH diagnosis onwards she was hospitalized in an intensive care unit. A sternal fracture occurred seven months after LDH due to coughing and osteoporosis. Finally, our patient died eight months after LDH diagnosis while on the high urgency waiting list for lung transplantation because of an unmanageable pulmonary infection with massive pulmonary bleeding. She had spent three weeks on extracorporeal membrane oxygenation (ECMO) before she died. The fact that she temporarily fell from the high urgency list a month before her death because of improving partial CO_2 _values on blood gas analysis may have also played a role in the disease process, as it tremendously destabilized her mood and raised fears she may not get a transplant organ.

## Discussion

Though the observation of LDH in CF has so far not been the clinical focus of the literature, and though the increased rate of LDH might also be coincidental, this special association seems clinically plausible. In a population-based epidemiological study of persons older than 30 years of age, the prevalence of radiologically-proven LDH with typical clinical symptoms was found to be 1.9% in men and 1.3% in women, with lower percentages in the age cohort of 30 to 44 years of age (men 1.0%, women 1.0%) [8]. At the age of about 20 years (the approximate age of our three patients at diagnosis) and in adolescence in general, LDH is extremely rare. This age distribution was also observed in people presenting to hospital for LDH surgery [9]. Pediatric cases only represent a marginal proportion (0.5% to 6.8%) of all LDH [10]. We hypothesize that the prevalence of 6.5% in our clinic population is due to CF-specific characteristics. Back pain in patients with CF is primarily of a musculoskeletal origin [11]. Frequent coughing can cause muscle splinting and musculoskeletal pain syndromes. The more the disease progresses, the more patients suffer from chronic coughing, often in spite of still-sufficient lung function parameters. Epidemiological studies clearly hint at the association of chronic cough and herniated lumbar inter-vertebral disc or sciatica due to widespread spondylotic changes of the lumbar spine [12]. Due to malnutrition and malabsorption syndromes compression fractures can occur in patients with osteoporosis as a result of the mechanical power of coughing, with peak cough expiratory flow rates ranging up to 700L per minute [13]. Patient 3 had a LDH with neurological deficits (paralytic symptoms) about six months before her death as a result of respiratory failure. A neurosurgical decompression operation was not performed as a result of the bad lung function. The accelerated process of lung deterioration was thought to be influenced by the limited ability to perform adequate respiratory therapy following the LDH. For example, it was not possible to perform reflectory respiratory treatment to its full extent, as well as some mucus mobilization maneuvers involving intensified compression techniques during assisted autogenous drainage and jumping on a trampoline or a pezzi ball. Opioids may also interfere with mucus drainage by a reduction of respiratory power, especially in higher doses. In addition to the physical aspect, the psychological burden on the patients with CF is extremely complex [14]. Patients with CF suffer from a chronic disease, inevitably leading to premature death respective to a high degree of morbidity. A reduced quality of life, ineffective coping strategies [15] and physical impairments increase the risk for anxiety and chronic depression in patients with CF [16]. Chronic painful physical conditions can increase the severity and duration of a depressive mood [17]. *Vice versa*, it is well documented in the literature that psychological factors play a key role in the pain perception process and the chronification of back pain [18]. The resulting chronic muscular imbalance and pathological muscular hypertension may be the basis for disc protrusion and disc rupture. Additionally, studies based on clinical [19] and experimental [20] data show that the risk for LDH is significantly increased in cases with a positive family history.

## Conclusions

LDH can lead to a vicious cycle for patients with CF as it may promote pulmonary infections and may decrease life expectancy. This underlines the need to investigate patients correctly and operate if necessary.

## Consent

Written informed consent was obtained from the patients and the patient's next-of-kin for publication of this manuscript and any accompanying images. A copy of the written consent is available for review by the Editor-in-Chief of this journal.

## Competing interests

The authors declare that they have no competing interests.

## Authors' contributions

CD wrote the article, and conceived of the study in cooperation with AV. HW gave psychological and MS radiological advice. MS was also involved in the diagnostic process as neurosurgeon. AG was involved in the therapeutic process of our patients as the head of the pulmonology department, and SB as the head of the pediatric department. All authors read and approved the final manuscript.
